# Weight Management Processes and Individual Differences: A Validation Study of P-Weight and S-Weight in Farsi

**DOI:** 10.34172/aim.33513

**Published:** 2025-03-01

**Authors:** Zahra Gohari Dezfuli, Minoo Hasan Rashedi, Mina Araminejad, Keyvan Karimi, Ensieh Sadat Mansouri, Tohid Seif Barghi, Amir-Hossein Memari

**Affiliations:** ^1^Sports Medicine Research Center, Neuroscience Institute, Tehran University of Medical Sciences, Tehran, Iran; ^2^Department of Clinical Nutrition, School of Nutritional Sciences and Dietetics, Tehran University of Medical Sciences, Tehran, Iran; ^3^Department of Nutrition, School of Public Health, Iran University of Medical Sciences, Tehran, Iran; ^4^Department of Epidemiology and Biostatistics, School of Public Health, Tehran University of Medical Sciences, Tehran, Iran; ^5^Department of Internal Medicine, School of Medicine, Sina Hospital, Tehran University of Medical Sciences (TUMS), Tehran, Iran

**Keywords:** Change strategies, Motivation, Personalized nutrition,, Processes, Readiness

## Abstract

**Background:**

The obesity epidemic is a growing public health concern, making weight management a crucial aspect of overall health and well-being. Indeed, effective tools to facilitate behavior change are essential for achieving long-term success in managing weight. This study aimed to validate the Farsi versions of the S-weight and P-weight questionnaires to support personalized weight management by assessing specific aspects of psychological readiness, including motivation, self-regulation, emotional reappraisal (EmR), and environmental restructuring (EnR).

**Methods:**

A cross-sectional study using self-administered questionnaires was conducted. The study included 455 adults aged 17–65, excluding those undergoing invasive weight-loss interventions. The measured variables included EmR, weight consequence evaluation (WCE), weight management actions (WMA), and EnR, which were assessed using structured Likert-scale questionnaires. Exploratory and confirmatory factor analyses were performed, with reliability evaluated via Cronbach’s alpha and intraclass correlation coefficient (ICC). Statistical significance was set at *P*<0.05.

**Results:**

The questionnaires showed strong validity and reliability (KMO=0.91; Bartlett’s test χ^2^=3999.75; *P*<0.001). Overweight and obese participants scored significantly higher in change processes than normal-weight participants (*P*<0.001).

**Conclusion:**

The validated instruments provide a reliable means of tailoring weight management strategies based on psychological readiness, potentially improving long-term outcomes.

## Introduction

 The global obesity epidemic represents a significant public health challenge. Rising obesity rates are particularly concerning in low- and middle-income countries, where limited resources, economic challenges, and cultural attitudes further complicate effective weight management. These challenges highlight the urgent need for sustainable strategies to address this growing crisis.^1^ While behavioral treatments such as diet and exercise may result in short-term weight loss, maintaining these results over the long term is often challenging, with weight regain being a common issue.^2,3^

 Maintaining weight loss can be challenging, as the body naturally seeks to stabilize its weight according to genetic and environmental factors. Approximately 95% of people regain weight within five years after dietary, drug, or behavioral treatments. This trend highlights the limited effectiveness of universal dietary interventions and emphasizes the growing shift toward personalized nutrition, recognizing that one size does not fit all.^4-6^ The limitations of universal approaches underscore the importance of psychological readiness and individualized strategies in achieving long-term weight management success.

 Personalized nutrition tailors dietary advice to an individual’s unique biological, psychological, and environmental characteristics, offering a more effective strategy for sustainable weight management.^7^ Moreover, psychological factors such as motivation, self-efficacy, and information processing significantly impact how individuals make food choices and adhere to dietary recommendations.^8^ Numerous theories focus on different aspects of behavioral change.^9,10^ Achieving and maintaining a healthy weight through behavioral changes requires effective treatment methods.^11,12^ Among these, the transtheoretical model of change, which involves evaluating stages of change and processes to modify behavior, is the most notable theory.

 Since the 1980s, the Transtheoretical Model (TTM) has been instrumental in promoting changes in health-related behaviors, such as smoking cessation, and more recently, in weight management. To operationalize TTM in weight management, two specific questionnaires (the S-weight and P-weight tools) have been developed. These instruments are designed to measure the steps and processes of change, providing a structured approach to assess readiness and strategies for weight management.^3,13^ A 2024 study by Gereklioglu further supported the effectiveness of TTM in managing obesity and overweight in adults.^14-17^ Behavior change in TTM unfolds in five stages: pre-contemplation, contemplation, preparation, action, and maintenance. Throughout these stages, individuals utilize strategies such as identifying their problem and taking steps to solve it. The four processes of change, including emotional reappraisal (EmR), weight consequence evaluation (WCE), environmental restructuring (EnR), and weight management actions (WMA), help people modify behaviors related to weight management.^3^

 Examining individuals in the UK and Spain revealed how the S-weight tool is linked to processes of change. These findings suggest that the P-weight and S-weight tools could be valuable for customizing weight management interventions.^3,18^ Understanding people’s readiness to change can greatly help weight loss efforts. Research has shown that adolescents struggling with obesity require additional support to modify their behaviors effectively.^19^ Moreover, tools such as specialized questionnaires have been validated to tailor weight loss interventions by assessing individual motivational stages and cognitive processes. This approach enhances the customization of weight management strategies based on personal readiness and psychological factors, improving the likelihood of successful outcomes.^20^

 However, despite advancements in behavioral and personalized treatments for obesity, there is a significant research gap in developing regions, particularly in the Middle East. These gaps include limited access to validated tools for weight management and lack of studies examining how processes of change relate to BMI or stages of change in diverse populations. This study aims to address these gaps by validating the P-weight and S-weight tools in the Farsi speaking-population for weight management. Furthermore, the study aims to provide insights into how processes of change vary across BMI classes or different stages of change.

## Materials and Methods

###  Participants and Data Collection

 This cross-sectional study involved 455 adults aged 17‒65 years from three nutrition and dietetics clinics in Tehran. The participants were using noninvasive weight management methods; patients with morbid obesity or those considering bariatric surgery were excluded. These individuals were excluded because their need for specialized and invasive interventions differs from the noninvasive, behaviorally focused strategies targeted in this study, which could have introduced variability and impacted the validity of the findings. Data were collected through online questionnaires between November 2023 and March 2024. All participants completed the S-weight and P-weight questionnaires once, while a subset of 70 participants retook these questionnaires four weeks later to assess reliability.

###  Measurements

 The S-weight questionnaire outlines the five stages of weight change according to the transtheoretical model. Pre-contemplation is the stage where there is no intention to change behavior in the next six months, and the individual is unaware of weight issues. Contemplation involves recognizing weight problems but not yet taking action. Preparation marks the phase where the individual starts to make changes within the next month, despite past failures. Action reflects efforts to change behavior over the past 6 months, and Maintenance focuses on sustaining new behaviors and preventing relapse.^19^

 The P-weight questionnaire assesses four processes of weight change using 34 questions with a 5-point Likert scale: EmR measures the emotional impact of weight and weight-management techniques; WCE examines the perceived negative effects of being overweight; WMA evaluates specific behaviors related to weight regulation; and EnR assesses changes in surroundings to support weight management. Scores are based on agreement levels, summed for each process, and scaled from 0 to 100, with higher scores indicating greater use of the process. Scores above 50 reflect high use, whereas scores below 50 indicate low use.^4^

###  Procedure

 In the initial phase, we aimed to assess the construct and cultural validity of the S-weight and P-weight questionnaires through an expert panel review, followed by exploratory factor analysis (EFA) and confirmatory factor analysis (CFA). The questionnaires were translated and back-translated. An expert panel then compared the translated and back-translated versions, discussing and confirming the final versions. The finalized questionnaires were then used in the subsequent steps. The data gathered from the questionnaires were subsequently randomly divided into two groups for analysis. EFA was conducted on the first group, whereas CFA was performed on the second group. Both groups were similarly distributed in terms of age, sex, and BMI (Table 1).

**Table 1:** Distribution of the Basic Characteristics of the Two Samples

**Variable**	**The First Half ** **(** ***n*** **=227)**	**The Second Half (** ***n*** **=228)**	***P*** ** Value**
Sex (female%)	68.2%	67.4%	0.85
Age (mean ± SD)	30.1 ± 10.6	30.7 ± 10.7	0.83
Weight (mean ± SD)	69.2 ± 16.5	70.3 ± 15.5	0.43
Height (mean ± SD)	167.1 ± 11.1	168.0 ± 9.1	0.46
BMI (mean ± SD)	24.9 ± 8.8	24.7 ± 4.3	0.39

N, number of participants in each category, SD, standard deviation.

 Additionally, 70 participants completed and refilled the finalized version of the questionnaires for the reliability assessment. Cronbach’s alpha and the mean of the overall correlation of items were used to examine the reliability of the items and the internal consistency of each retained factor in both samples. The intraclass correlation coefficient (ICC) was used to assess the test-retest reliability of the weight change questionnaire over four weeks.^4^ In addition, the Kendall rank correlation was used to evaluate the test‒retest reliability of the single-item stage of the weight change questionnaire. An ICC and Cronbach’s alpha greater than 0.70 are considered to indicate acceptable reliability.^21^

 In the second phase, we examined the processes of change according to individual differences. We compared the average values of the P-weight components among different BMI groups: normal, overweight, and obese. Additionally, we compared the average values of the P-weight components across different stages of weight change, including pre-contemplation (PC), contemplation (C), preparation (Prep), action (A), and maintenance (M). We hypothesized that participants would use the process of change strategies differently based on differences in their BMI category.

###  Statistical Analyses

####  First Phase

 The statistical software Stata, version 18, was used for the stages of data analysis. The adequacy of the sample size for the EFA was assessed according to the Kaiser‒Meyer‒Olkin (KMO) criterion (KMO > 0.50 acceptable),^22^ and a significant correlation between the items was established by Bartlett’s test for sphericity (*P* < 0.05). EFA was conducted on the first half of the data (n = 227) using principal component analysis (PCA). Then, oblimin rotation with eigenvalues greater than 1 and a factor loading greater than 0.35^23^ was applied to assign each item to one of the four dimensions. In the next step, CFA was applied using the AMOS 17.0 statistical package to confirm item loading in each latent factor in the second sample of participants (n = 228). Since the data were not normally distributed, the unweighted least squares (ULS) method was used to assess the goodness-of-fit of the CFA models. Based on this method, models with flow indices and critical values assume a fitted model: goodness-of-fit index (GFI ≥ 0.90), adjusted goodness-of-fit index (AGFI ≥ 0.90), standardized goodness-of-fit index (NFI ≥ 0.90), standardized root mean square residual (SRMR ≤ 0.08) and χ2/df or CMIN/df < 3).^24^

####  Second Phase

 In the second phase, we aimed to cross-culturally adapt the S-weight and P-weight questionnaires for weight management and to compare individuals’ mindsets according to their BMI in each stage of the process of change.

 We conducted a one-way ANOVA with *post-hoc* comparisons to compare the mean values of the P-weight components among the various BMI groups, including normal, overweight, and obese groups. Additionally, the mean values of the P-weight components were compared across different stages of weight change, including pre-contemplation (PC), contemplation (C), preparation (Prep), action (A), and maintenance (M). Trends in the mean P-weight values at different stages of weight change were also plotted in a line graph.

## Results

###  Participants

 Data were obtained from 455 participants aged between 17 and 60 years, with a mean age of 30.2 ± 10.6 years. A total of 309 participants were women, and the rest were men. On average, the women in this study were younger than the men (mean age: women = 29.6, men = 31.4). The total data were split into two random samples with similar distributions in terms of age, sex, body weight, and height. There were no significant differences between the participants in the first half of the data and those in the second half of the data (Table 1 for demographic information). Indeed, the two samples were so similar that EFA and CFA could be applied separately.

###  Explanatory Factor Analysis

 The factor test results indicate that the sample size of the first half of the data was sufficient to apply PCA for factor extraction of the 34 items on the weight change processes questionnaire (P-weight) (KMO = 0.91, Bartlett’s test χ^2^ = 3999.75, *P* < 0.001). Four factors were rotated, followed by PCA, which explained 61.12% of the variance across the 34 items. Based on the content of the items and the loading of the individual components, the factors were named, and the corresponding items were assigned. Thirteen items were categorized as emotional re-evaluations (EmRs) as the first factor. All 13 items had acceptable factor loadings ranging between 0.36 and 0.77. The second factor comprised 7 items with factor loadings between 0.35 and 0.74 related to WMA. Five items with factor loadings between 0.43 and 0.65 were defined as environmental restructuring (EnR), and nine items loaded into the WCE category were defined as the last factor (Table S1 for EFA results in detail).

###  Internal Consistency

 The ICC was calculated to analyze the test-retest reliability of the 34-item P-weight questionnaire. The ICC was 0.92, with a 95% confidence interval (95% CI: 0.88–0.96) (see Table 2 for reliability analysis). Furthermore, the reliability for the single-item stage of weight change was 0.93 (95% CI: 0.86–0.97). Both questionnaires had good reliability over 14 days. Cronbach’s alpha was reported for each of the four components and associated items to assess internal consistency (reliability). In the first half of the data (n = 227), Cronbach’s alpha values were 0.89, 0.74, 0.85, 0.79, and 0.90 for the EmR, EnR, WCE, WMA, and total items, respectively. The reliability of the factors in the second half of the data (n = 228) also showed good consistency (EmR; 0.87, EnR; 0.72, WCE; 0.88, WMA; 0.80 and total; 0.93). The total inter-item correlation between the items was acceptable (ranging from 0.53 to 0.61 and 0.46 to 0.67, respectively) in samples 1 and 2 (Table 3 for reliability detail).

**Table 2:** Test-Retest Reliability of the Questionnaires Used in the Study

**Questionnaire**	**ICC**	**95% CI**	***P *** **Value**
Process of weight change	0.92	(0.89‒0.94)	< 0.001
Stage of weight change	0.77^a^	(0.73–0.84)	< 0.001

ICC, Interclass correlation coefficient; ^a^ Kendall’s tau correlation.

**Table 3:** Interitem Correlation and Reliability of the Four Subdomains of the 32-Item Questionnaire

**Factor**	**No. of Items**	**The First Half (** ***n*** **=209)**		**The Second Half (** ***n*** **=246)**	
		**Cronbach's Alpha**	**Item-Total Correlations**	**Cronbach's Alpha**	**Item-Total Correlations**
EmR	12	0.89	0.60	0.87	0.52
EnR	5	0.74	0.53	0.72	0.46
WCE	9	0.85	0.61	0.88	0.67
WMA	6	0.79	0.58	0.80	0.54
Total	32	0.90	0.44	0.93	0.40

EmR, Emotional re-evaluation; WMA, Weight management actions; EnR, Environmental restructuring; WCE, Weight consequences evaluation. ** *P* < 0.001 and *P < 0.05

###  Confirmatory Factor Analysis

**Figure 1:**
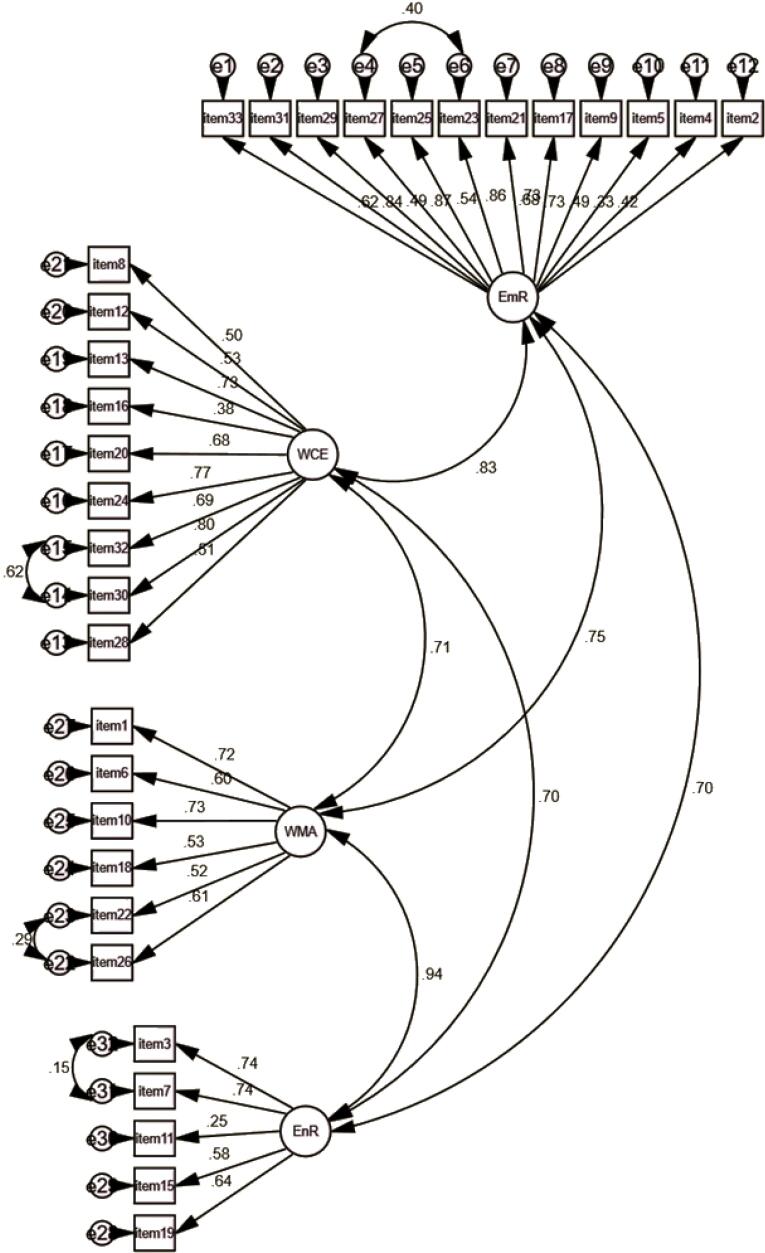
Confirmatory Factor Analysis Results for 32 Items

 The second half of the data (n = 228) was used to confirm the extraction components and the corresponding items. The ULS method revealed that all items for the previously identified components loaded with acceptable weights (greater than 0.35), except one item in the EmR factor (‘I am committed to losing weight’) with a factor loading below 0.35 (0.25), and one item in the WMA factor (‘I have learned to control my appetite’) with a low factor loading (0.24). Two of these items were removed from the related factors to improve the model’s goodness-of-fit (see the CFA fitness indices in Table 4). In addition, the content of these two items was evaluated and discussed by experts in order to find a consensus for the deletion. In this model, all 32 items loaded with an acceptable weight in four first-order factors were between 0.32 and 0.87. The correlation between the four factors was also between 0.70 and 0.94 (Figure 1 for CFA graph). Both models had an acceptable fit, even though Model 2 (32 items) was better adapted to the sample (Table 4 for CFA fitness indices).

**Table 4:** Goodness-of-Fit Indices for Assessing CFA Models

**Model **	**CMIN/df**	**GFI**	**AGFA**	**NFI **	**SRMR **
1	3.7	0.957	0.951	0.946	0.057
2	2.9	0.968	0.963	0.960	0.056

CMIN, Chi-square test; d.f., Degrees of freedom; GFI, Goodness-of-fit index; AGFI, Adjusted goodness-of-fit index; NFI, Normed fit index; SRMR, Standardized root mean square residual Model 1: 34 items remain. Model 2: item 14 and 34 with factor lading 0.24 and 0.25 were deleted.

###  P-weight and Individual Differences

**Figure 2:**
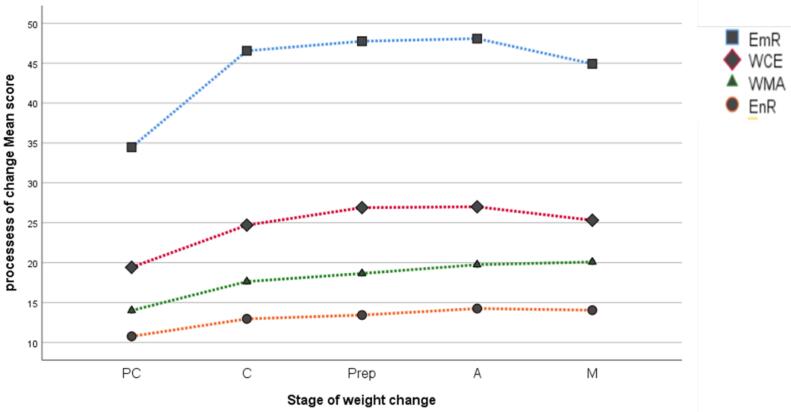
Mean Scores of the Processes of Weight Change Across Different Body Weight Groups. EmR, Emotional re-evaluation; WMA, Weight management actions; EnR, Environmental restructuring; WCE, Weight consequences evaluation; PC, Pre-contemplation; C, Contemplation; Prep, Preparation; A, Action; M, Maintenance

**Figure 3:**
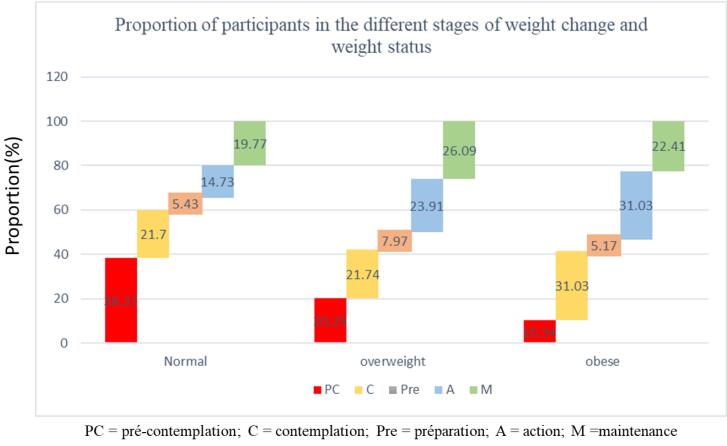
Distribution of People with Different Stages of Weight Change by Weight Status

 The mean score of the P-weight questionnaire was compared across the participants with different weight levels (normal weight, overweight, and obese). The results of the one-way ANOVA indicated a significant difference between the three groups regarding the mean score of the four dimensions. The mean EmR scores for the normal weight, overweight, and obese participants were 40.1, 46.0, and 48.7, respectively. A post-hoc Scheffe comparison revealed that overweight and obese participants had significantly greater mean EmR scores than normal-weight participants (*P*< 0.001). Compared with the overweight and obese groups, the normal-weight group had lower mean scores in all dimensions and overall assessments (*P*< 0.001). In addition, the mean scores of the WCE and total scales were significantly lower in the overweight group than in the obese group (*P*< 0.001) (Table 5 for P-weight difference across BMI status). In addition, the mean P-weight (four dimensions) was compared across the five stages of weight change (pre-contemplation (PC), contemplation (C), preparation (Prep), action (A), and maintenance (M)) reported by the participants. In all dimensions, the mean scores increased from stage 1 (PC) to stage 4 (A) and then slowly decreased in the last stage (M) (*P*< 0.001) (Figure 2 and Table 6 for P-weight difference across Stages of weight change). (Figure 2 and Table 5). Regarding weight status, normal weight individuals were more represented in the PC phase of weight change than overweight and obese individuals (*P*< 0.05). In the M phase, on the other hand, overweight people were more represented than obese and normal-weight people (26.09%, 19.77% and 22.41% respectively) (*P*< 0.05). Obese people were more represented in the action phase (A) of weight change than others (*P*< 0.05) (see Figure 3 for more details).

**Table 5:** Comparison of the Mean Score of Weight Change Processes Across Different Body Weight Groups

**Weight Status**	**Normal Weight** **(N=259)**	**Overweight** **(N=138)**	**Obeses** **(N=58)**	**F Statistic**	***P *** **Value**	**Boefroni Comparison**
**Factor**	**Mean (SD)**	**Mean (SD)**	**Mean (SD)**			
EmR	40.1 (9.6)	46.0 (8.2)	48.7 (7.4)	33.08	< 0.001	A < B^**^, A < C^**^, B < C
EnR	12.0 (4.2)	13.5 (3.7)	14.8 (3.9)	14.51	< 0.001	A < B^*^, A < C^**^, B < C
WCE	20.7 (6.4)	26.1 (6.8)	32.6 (7.1)	87.65	< 0.001	A < B^**^, A < C^**^, B < C^**^
WMA	16.7 (5.3)	18.4 (4.3)	19.6 (4.8)	10.93	< 0.001	A < B^*^, A < C^**^, B < C
Total	89.5 (21.7)	103.9 (18.1)	115.8 (18.8)	50.22	< 0.001	A < B^**^, A < C^**^, B < C^*^

N, number of participants in each category, SD, Standard deviation, A, Normal weight, B, Overweight, C, Obese, EmR, Emotional re-evaluation; WMA, Weight management actions; EnR, Environmental restructuring; WCE, Weight consequences evaluation. ** *P* < 0.001 and * *P* < 0.05.

**Table 6:** Comparison of the Mean Score of Processes of Weight Change Across Weight Management Stages

**Weight Status**	**Weight Management Stages**					***P*** ** Value**	**Boefroni Comparison**
	**PC** **(N=133)**	**C** **(N=104)**	**Pre** **(N=28)**	**A** **(N=89)**	**M** **(N=100)**		
**Factor**	**Mean (SD)**	**Mean (SD)**	**Mean (SD)**	**Mean (SD)**	**Mean (SD)**		
EmR	34.4(9.8)	46.5(6.5)	47.7(7.8)	48.0(6.7)	44.9(6.9)	< 0.001	PC < C^**^, C < Pre^**^, pre < M,A > M
EnR	10.7(3.8)	12.9(3.6)	13.4(4.0)	14.2(3.7)	14.1(4.22)	< 0.001	PC < C^**^, C < Pre, pre < M,A > M
WCE	19.4(6.4)	24.7(7.1)	26.9(7.5)	27(7.7)	25.3(7.7)	< 0.001	PC < C^**^, C < Pre^*^, pre < M,A > M^*^
WMA	14(4.8)	17.6(4.5)	18.6(3.3)	19.7(3.9)	20.1(4.4)	< 0.001	PC < C^**^, C < Pre^*^, pre < M,A > M
Total	78.6(21.5)	101.8(17.6)	106.7(16.1)	109(17)	104.2(18.4)	< 0.001	PC < C^**^, C < Pre^*^, pre < M^*^,A > M^*^

N, Number of participants in each category, SD, Standard deviation, PC, Pre-contemplation; C, Contemplation; Prep, Preparation; A, action; M, maintenance; EnR, Environmental restructuring; EmR, Emotional re-evaluation; WMA, Weight management actions; SR, Supporting relationship; WCE, Weight consequences. evaluation. ** *P* < 0.001 and * *P* < 0.05.

## Discussion

 Our study successfully validated the Farsi versions of the S-weight and P-weight questionnaires to assess weight management strategies. The analyses confirmed that both versions possess high reliability, as evidenced by robust ICC and strong Cronbach’s alpha scores.^25,26^ Even though two out of the 34 items did not achieve satisfactory factor loadings in the CFA, both the 32-item and 34-item models showed excellent reliability and demonstrated similar goodness-of-fit indices.^27^ Furthermore, the structural validity of our questionnaires was consistent with the findings of studies conducted in the UK, Brazil, and Spain, which reported comparable fit indices in validating these tools (GFI: 0.974, 0.988 and 0.960).^3,20,28^

 This study adapted the P-weight questionnaire to account for participants’ BMI classifications and the specific strategies applicable to each stage of weight management. When comparing the mean scores of participants with different weight categories (namely normal weight, overweight, and obese) significant differences emerged across the questionnaire’s various dimensions. Notably, those in the overweight and obese groups exhibited higher scores in the ‘emotional re-evaluation’ category, suggesting a greater likelihood of pursuing weight loss for emotional motivations. In contrast, participants within the normal-weight range consistently reported lower mean scores across all dimensions of the P-weight questionnaire.^18^ Additionally, the ‘WCE’ scores were notably lower in the overweight group compared to the obese group. In addition, the mean scores of the weight consequences evaluation were significantly lower in the overweight group than in the obese group. On the other hand, individuals with higher BMIs acknowledge the need for change, reevaluate personal values and behaviors, and comprehend the advantages of weight loss. Higher BMI groups may experience heightened awareness of health risks associated with obesity, including chronic diseases such as diabetes and cardiovascular disorders. This awareness can amplify the emotional impact of being overweight, potentially increasing the emphasis on EMR as a strategy for weight management. Sociocultural factors may also play a role, particularly the stigma associated with obesity, which is more pronounced in individuals with higher BMI. Weight stigma has been shown to negatively impact psychological well-being, leading to stronger emotional reactions and a heightened focus on the consequences of being overweight. They are more likely to change the environment to lose weight. Indeed, the increased health risks in obese individuals are likely to enhance their motivation, leading to more intensive involvement in these processes.^29-31^

 The distribution of participants in different BMI categories across weight management stages yields further valuable insights. A significant portion of obese participants actively engaged in weight management (action stage), which indicates participants with higher BMI are more likely to be in the action or maintenance stages, reflecting a greater recognition of the need for ongoing weight management efforts (Figure 3). Another assessment that we performed in this study was the comparison of P-weight variables across the five stages of weight change.^29,32^ Additionally, we observed considerable progress in the use of change strategies as individuals moved from the pre-contemplation stage to the action stage. For example, individuals in the action stage focus more on re-evaluating their environment and emotions compared to those in the pre-contemplation group. On the other hand, individuals in the maintenance group engage in more self-evaluation compared to those in the action group. This subdomain is different from other subdomains, such as WMA, EMR, and EnR.^29,32^

 Our results align with previous research, such as Ceccarini’s review, which demonstrated that the S-weight and P-weight questionnaires effectively identify an individual’s stage within TTM of behavior change for weight control. These questionnaires are also instrumental in evaluating both explicit and implicit behaviors related to weight management, and they can detect resistance to change, particularly in the early pre-contemplation stage. Studies by DiClemente et al and Prochaska et al have underscored the finding that the processes measured by these tools are critical predictors of successful behavior change across different stages.^33,34^ Furthermore, Prochaska et al highlighted that a deeper understanding of various P-weight factors (such as environmental, interpersonal, and social influences) enhances the effectiveness of weight management strategies. This insight is consistent with our study, which found that engagement was higher in the action stage than in both pre-contemplation and maintenance stages, highlighting the importance of tailored motivational interventions.^34^ Andrés’s research findings showed psychometric properties of the P-weight as well, and declared the importance of internal changes such as BMI or the stage of change rather than external variables. They also indicated lower use of change processes in the PC stage than in later stages. Our study supports these findings by demonstrating that environmental, psychological, and personal intentions could be affected and modified by individual differences. This thorough evaluation facilitates the development of more personalized and potentially more effective targeted weight management strategies.^35,36^

 Notably, some previous studies have focused primarily on populations preparing for bariatric surgery, which is a great confounding factor because presurgical behavioral adjustments may influence their response. Indeed, their motivation and behaviors can be affected by the anticipated surgical intervention.^38,39^ In contrast, our study included a different population; thus, we could evaluate other internal factors that affect weight change strategies.

 To address the practical implications, the findings indicate that as individuals move through the stages of change, their engagement with different processes of change varies. This highlights the importance of stage-specific interventions in weight management programs, aligning with the TTM framework. The results also suggest that interventions should focus more on supporting individuals in the Maintenance stage to sustain their behavioral changes, as the use of change processes tends to stabilize or decrease in this stage.^39-41^ In Iran, cultural preferences related to beauty standards and food practices might shape motivations for weight loss and affect the success of interventions. For example, social pressures to maintain a particular body image may increase the importance of weight management, but the availability and acceptance of certain food types, as well as sedentary lifestyles, might present barriers.

 This study was not without limitations. First, the nonrandom sampling procedure limits the generalizability of the results to the broader Iranian population or other groups with different characteristics. For instance, the overrepresentation of certain BMI categories could have skewed the results toward highlighting processes of change more relevant to those groups, potentially underestimating or overemphasizing the applicability of TTM constructs in other populations. Additionally, longitudinal studies are needed to assess the long-term effectiveness of TTM-based interventions in sustaining weight loss and behavioral changes.

## Conclusion

 The findings of this study demonstrate that the Farsi versions of the S-weight and P-weight questionnaires exhibit high internal consistency, robust construct validity, and excellent test-retest. These results establish the questionnaires as reliable and valid tools for assessing psychological readiness in weight management processes based on TTM. Insights from this study facilitates the development of weight management programs tailored to address these specific strengths and weaknesses, allowing for targeted interventions that focus on the necessary motivational and behavioral triggers, which are particular for each individual.

## Supplementary Files

Supplementary file 1 contains Table S1.
