# Supplementary material for: Weight Management Processes and Individual Differences: A Validation Study of P-Weight and S-Weight in Farsi
Source: Arch Iran Med. 2025 Mar 1;28(3):162–70. doi: 10.34172/aim.33513 (PMC12038800; doi:10.34172/aim.33513)
Supplement: Supplementary file 1 — contains Table S1. [file aim-28-162-s001.pdf]

Arch Iran Med. March 2025;28(3):162-S1

10.34172/aim.33513

Supplementary file 1

Table S1. Factor loading of items resulting from principal component analysis.

Items	Communalities	Factor lading			
		EmR	WMA	EnR	WCE
I'm not happy with my current weight	0.33	0.58			
I think I should eat food with less fat	0.75	0.48			
I feel good when I am able to control my eating habits	0.52	0.59			
When I lose weight, I feel proud of myself	0.28	0.71			
I have someone who listens to me when I need to talk about my being overweight	0.30	0.75			
I now realize I have a weight problem	0.46	0.61			
Society's view of obese people affects me emotionally	0.76	0.36			
Being overweight makes me feel bad	0.32	0.74			
If I lost weight, I would feel better about myself	0.47	0.64			
If I lost weight, I would be happier	0.53	0.48			
I am worried about gaining more weight	0.32	0.77			
I feel guilty when I overeat	0.43	0.63			
I am committed to losing weight	.56	0.37			
I look for information about the types of food which could help me to lose weight	0.46		0.43		
I tell myself positive things to avoid overeating	0.53		0.56		
When I truly want to eat, I do activities to avoid it	0.43		0.67		
I have learnt skills that reduce my desire to eat (e.g. distracting myself)	0.41		0.74		
I have learned to control my appetite	0.70		0.35		
I avoid buying high-calorie food	0.47		0.64		
To avoid overeating I prefer eating at home or cooking my own food	0.54		0.48		
I try to put food away to avoid nibbling	0.46			0.60	
I try not to have food in sight	0.40			0.63	
There are no snack foods in my fridge or cupboards	0.69			0.51	
I avoid places where people eat a lot	0.44			0.65	
When I am on a diet1 I avoid eating with people who I overeat with	0.65			0.43	
My weight restricts my relationships	0.31				0.78
My current weight makes my daily life difficult	0.33				0.74

Losing weight would help me improve my relationships with others	0.38				0.61
My family and friends are worried about my weight	0.32				0.78
Most of my health problems are due to my being overweight	0.44				0.63
I am aware that there are more and more people who encourage me to lose weight	0.33				0.51
My family and friends praise me for not overeating	0.60				0.52
My family and friends congratulate me when I manage to lose weight	0.28				0.71
People around me support me in trying to lose weight	0.30				0.75
**Eigenvalue**		**11.34**	**3.39**	**2.09**	**1.51**

EmR = Emotional re-evaluation; **WMA** = weight management actions; **EnR** = environmental restructuring; **WCE** = weight consequences evaluation
